# Replication studies hold the key to generalization

**DOI:** 10.1038/s41467-022-34748-x

**Published:** 2022-11-16

## Abstract

Rigorous, robust replication and generalization studies present a significant investment into the quality of the research record, the foundation on which new research is built. Communications Psychology and the Human Behaviour Team at Nature Communications are inviting submissions of replication studies in psychology and the human behavioural sciences.

Replication studies, in which scientists closely match the study setup and analysis reported in previous work to test whether they find the same results, rose to fame in psychology with an influential multi-lab study led by Brian Nosek in 2015 (https://www.science.org/doi/10.1126/science.aac4716). The work consisted of the replication of 100 studies, of which 97 studies had reported a significant result in the original publication. In the replication, only 35 resulted in statistically significant results in the same direction as the original work. The work brought to the fore a concern that had been gaining momentum over previous years: that the way scientific research is conducted, from the initial study plan to the final publication, leaves the door wide open for unreliable studies to be published and for spurious results to be accepted as genuine effects.

Working hand-in-hand with many other initiatives aimed at improving the quality of scientific research, replication studies have played a fundamental role in setting the scientific literature on a stronger footing. They unquestionably continue to play a fundamental role in increasing trust in research, by demonstrating what effects reliably hold, and by revealing what previously established effects should not be taken for granted or require further scrutiny.

Although replication studies are by no means the only highly-powered, rigorous large-scale studies conducted today, they have no doubt contributed to making this type of multi-lab research popular. They also led to a better understanding of the nature of studies that produce robust inferences in psychology, and shed light on underappreciated pitfalls in research practices. The knock-on effect of these valuable lessons is a positive influence on the field at large.

One of the tricky questions that this type of research has prompted is that of standardization and generalization- the wider meaning of how results relate to the exact matching of study conditions. Reviewing the implementation of ostensibly the same paradigm, has in some instances revealed an unexpected lack of standardization, with vast variation between different implementations of a task (see https://flexiblemeasures.com/ for examples). One the one hand, this variation can be problematic, as it raises the question whether different researchers on a topic are measuring—or indeed talking about—the same phenomenon. On the other hand, systematic comparisons of implementation conditions may explain contradictory findings^1^. Establishing the same pattern of behavior across different implementations may endow the replicated finding with more credibility - and an enlarged prospect of real-world relevance. Replication studies that not only closely reproduce an existing version of a study setup and analysis, but in addition test its generalizability to different versions (e.g., of a task) or new contexts (e.g., real-life interventions for lab-based studies) have a tremendous potential to deepen our understanding of what past results mean.

“Establishing the same pattern of behavior across different implementations may endow the replicated finding with more credibility—and an enlarged prospect of real-world relevance.”

Multi-lab replication studies that bring together many collaborators from different countries can also help to address another long-standing issue of psychological research: a fundamental lack of work that reflects global diversity. An overwhelming number of studies still rely on participants who are living in Global North/Western nations, who are often university students or university educated. Standard replication studies may demonstrate whether specific effects reliably co-occur in a population that closely matches the original sample. But if it is the aim of psychology to uncover universal effects or delineate their boundaries, what the field needs are generalization studies, regardless of where the original effect was first established^2^. Ultimately, this is also necessary to ensure that any intervention aimed at addressing a particular problem is designed keeping in mind the prevalence of the issue in each population.

Communications Psychology and the Human Behavior team at Nature Communications welcome both direct replication studies that aim to test the robustness of previously established effects and generalization studies that critically investigate the degree to which these effects hold across different settings and populations. The present call is specific to studies in psychology and closely neighboring fields, such as animal models of human cognition, behavioral or neuroeconomics, education, and (computational) psychiatry. The journals will prioritize peer-review of work that focuses on effects that are of continuing relevance to the discipline, but not yet convincingly supported by the literature. Submissions are welcome as standard research articles or in the Registered Report format and submissions in 2022 and 2023 will become part of a cross-journal special collection.

Submissions as Registered Reports should follow the journals’ standards detailed in the Nature Communications and Communications Psychology guides to authors. For submissions in the format of a standard research article preregistration will be considered favorably in the editorial evaluation. More information about preregistration policies at Communications Psychology and Nature Communications, as well as standards for statistical reporting, including for null results are available on the journals’ webpages.

We are looking forward to receiving your submissions as we see supporting this type of work as an investment in the future of psychology.
